# Teeth of Old Age

**Published:** 1896-10

**Authors:** 


ARTICLE II.

### TEETH OF OLD AGE.

BY. DR. BLACK.

It is a fact that the teeth become more dense and their specific gravity becomes greater from youth to old age; but this difference is not great. It is a difference that requires the finest powers to demonstrate. The increase in strenght is not very great. The difference between teeth is not very great, but it is certain. Follow those differences and you will see. Take the teeth of a young child, and you find average density; take the child in the teens and then in the twenties, and you find an increased density; then up to thirty there is slower increase, and from thirty to forty or forty-five the increase is very slight.

From forty-five to sixty the increase in density is greater again. This is the way in which this has developed itself in my experimental work. I understand that Tomes is now repeating this work. I expect that he will practically substantiate these results, though not precisely, for no two sets of teeth taken from among one hundred persons will give the same results. The difference in density of the teeth of the same person is almost as great as the differences in the teeth of one hundred persons.

Of course, the moment the pulp is dead the increase of density stops. This increase of density occurring in old age or in persons past middle age shows very plainly that the teeth require nutrition throughout life. In teeth that are worn down the pulp has receded, and the enamel has receded, and when it is worn away the strength of the dentin is impaired in proportion to the recession of the pulp. That wearing way of the teeth that we have come to regard as normal produces an abnormal condition of the tissues of the dentin. That tissue of the dentin has lost its vitality, fluids are admitted to the dentinal tubes, and that dentinal tissue becomes impaired and its strength is gradually lost in perfectly sound teeth. This is shown very clearly by experiments. Wherever we have a tooth that has lost its pulp and the vitality of the dentin is gone, and that tooth begins to show a discoloration, there we find that the strength of the dentin is impaired. When we come to test its strength, with the dynamometer, we find that the strength is impaired. We find a peculiar disposition of the enamel to chip of from the dentin. It is much more liable to be broken away. In the tooth that has its plug and its proper nutrition, this parting from the dentin is not observed. This difference became very marked in this class of experimental work, all of which goes to show the value of the dental pulp, not simply in youth but throughout life. The breaking away and the causes of that breaking away seem to be well shown in this class of experimentation. The pulp is important, not only in youth but continually thereafter. As a person grows older its importance may be diminished, but it is still important, and it seems to me, that if we can do anything to prevent the wearing down of the tooth, it becomes our duty to do so. We can build up the tooth with platina, thoroughly annealed and malleted, and we can make it much harder than hammered cast gold. We can make it stronger than the gold we put through our rolling-mills and make into plates.—*Items of Interest.*

